# High bone marrow angiopoietin-1 expression is an independent poor prognostic factor for survival in patients with myelodysplastic syndromes

**DOI:** 10.1038/bjc.2011.340

**Published:** 2011-08-30

**Authors:** C-L Cheng, H-A Hou, J-Y Jhuang, C-W Lin, C-Y Chen, J-L Tang, W-C Chou, M-H Tseng, M Yao, S-Y Huang, B-S Ko, S-C Hsu, S-J Wu, W Tsay, Y-C Chen, H-F Tien

**Affiliations:** 1 Division of Hematology, Department of Internal Medicine, National Taiwan University Hospital, College of Medicine, National Taiwan University, No. 7, Chung-Shan South Road, Taipei 10002, Taiwan; 2 Department of Pathology, National Taiwan University Hospital, College of Medicine, National Taiwan University, Taipei, Taiwan; 3 Tissue Bank, Center for Genomic Medicine, National Taiwan University, Taipei, Taiwan; 4 Department of Laboratory Medicine, National Taiwan University Hospital, College of Medicine, National Taiwan University, Taipei, Taiwan

**Keywords:** angiopoietin, vascular endothelial growth factor, myelodysplastic syndromes, prognosis

## Abstract

**Background:**

Angiogenic factors have an essential role in normal and pathologic angiogenesis. However, the clinical implication of angiogenic factor expression in myelodysplastic syndromes (MDS) remains unclear.

**Methods:**

In this study, we sought to investigate the prognostic impact of the expression of genes encoding angiopoietin-1 (*Ang-1*), *Ang-2*, the receptor *Tie2*, vascular endothelial growth factor-A (*VEGF-A*) and *VEGF-C* in the bone marrow (BM) in 208 patients with newly diagnosed primary MDS.

**Results:**

BM *Ang-1* expression was significantly higher in MDS patients, especially those with higher-risk subtypes, than in normal controls. With a median follow-up time of 32.9 months, the disease transformed to acute leukaemia more frequently in the patients bearing higher *Ang-1* expression than in those with lower expression (31.5% *vs* 18.6%, *P*=0.023). The MDS patients with higher *Ang-1* expression had shorter overall survival than those with lower expression (median 20.8±4.5 months *vs* 63.3±17.8 months, *P*<0.001). Multivariate analyses showed that higher *Ang-1* expression was an independent unfavourable prognostic factor for overall survival. There was no impact of the expression of other angiogenic factors on survival.

**Conclusion:**

BM *Ang-1* expression may serve as a new biomarker to predict clinical outcome in MDS patients.

Angiogenesis, the sprouting of new blood vessels from an existing vasculature (Cines *et al*, 1998), has a crucial role in the growth, dissemination and metastasis of solid tumours (Folkman, 1971; Ellis and Fidler, 1996). Several antiangiogenic drugs have been shown to be effective in various forms of cancers, such as bevacizumab in colon cancers and lung cancers (Kabbinavar *et al*, 2003; Hurwitz *et al*, 2004; Sandler *et al*, 2006). Recent studies suggest that the progression of haematologic malignancies, such as acute myeloid leukaemia (AML) (Aguayo *et al*, 1999; Hussong *et al*, 2000; Padro *et al*, 2000), is also strongly related to angiogenesis. Myelodysplastic syndromes (MDS) are a heterogeneous group of clonal haematopoietic disorders characterised by ineffective haematopoiesis (Mufti, 2004) and a propensity to transform to AML. Growing evidences indicate a key role of angiogenesis in the pathogenesis of MDS. Bone marrow (BM) microvessel density (MVD) as displayed by immunohistochemical (IHC) staining has been shown to be higher in MDS patients than in normal populations (Pruneri *et al*, 1999; Aguayo *et al*, 2000; Korkolopoulou *et al*, 2001; Alexandrakis *et al*, 2005). The studies regarding the correlation between BM MVD and the stage of MDS showed conflicting results; some reported higher MVD in high-risk MDS than in low-risk ones (Pruneri *et al*, 1999; Alexandrakis *et al*, 2005), while others did not find any difference among French–American–British (FAB) subgroups (Lundberg *et al*, 2006). The prognostic implication of BM MVD in MDS patients also remains controversial (Korkolopoulou *et al*, 2001; Alexandrakis *et al*, 2005; Lundberg *et al*, 2006; Madry *et al*, 2007).

Recently, the expression of angiogenic factors including vascular endothelial growth factors (VEGF) and angiopoietin (Ang)-Tie2 receptor family was studied to evaluate the role of these factors in normal and pathologic angiogenesis. The expression of Ang and VEGF correlates well with clinical features and outcome in patients with solid cancers (Toi *et al*, 1994; Salven *et al*, 1998; Tanaka *et al*, 2002; Chung *et al*, 2006). The studies concerning clinical implication of the expression of these factors in MDS are limited. The blood levels of VEGF were increased in MDS patients compared with normal controls (Aguayo *et al*, 2002), especially in those with advanced MDS (Brunner *et al*, 2002). The mRNA expression of *Ang-1*, *Ang-2* and the receptor *Tie2* in the BM was significantly higher in MDS patients compared with normal controls (Keith *et al*, 2007). Nevertheless, the case numbers of these studies were limited. In addition, the prognostic implication of the expression of these angiogenic factors remains investigational. In this study, we investigated RNA expression of genes encoding *Ang-1*, *Ang-2*, *Tie2*, *VEGF-A* and *VEGF-C* by real-time quantitative polymerase chain reaction (RQ-PCR) in a cohort of 208 patients with newly diagnosed primary MDS and correlated the results with clinical features and outcome of the patients. We found that *Ang-1* expression was an independent prognostic factor in MDS patients.

## Materials and methods

### Patients and samples

A total of 208 adult patients with newly diagnosed primary MDS according to FAB criteria at the National Taiwan University Hospital between January 1986 and December 2008 who had complete clinical data and available RNA for study were recruited. Twenty normal marrow donors were also enrolled for comparison. Expression of *Ang-1*, *Ang-2*, *Tie2*, *VEGF-A* and *VEGF-C* in the BM was determined at diagnosis. One hundred and seventy-seven (85.1%) patients received supportive care. The remaining 31 patients were treated with AML-directed intensive chemotherapy (*n*=8), allogeneic haematopoietic stem cell transplantation (*n*=18) or both (*n*=5). This study protocol was approved by the Institutional Review Board of the National Taiwan University Hospital and all patients gave their informed consent.

### Real-time quantitative polymerase chain reaction

BM mononuclear cells from 208 patients and 20 healthy transplantation donors were isolated and cryopreserved until use. Total RNA was extracted, reverse transcribed and amplified by RQ-PCR as previously described (Hou *et al*, 2008). Each sample was tested at least twice independently. The amount of the target genes was normalised to the housekeeping gene *RPLP0*. The copies of target gene were quantified only after successful amplification of the internal control, using standard curves derived from cloned plasmids. All data were presented as log ratio of the target gene/*RPLP0*. The sequences of primers and probes of these five angiogenic factors were listed in Supplementary Table 1.

### IHC staining for Ang-1 protein

We performed IHC to assess the Ang-1 protein expression in the BM biopsy specimens from 40 selected MDS patients, 20 having higher *Ang-1* mRNA expression and another 20, lower *Ang-1* mRNA expression. BM biopsies were collected and placed immediately into 10% buffered formalin fixative. The fixation time was between 6 and 24 h. The specimens were washed in tap water for 2– min and decalcified with Plank-Rychlo's solution (Muto Pure Chemicals Co., Ltd, Tokyo, Japan) in the specimen container. The decalcification time was between 90 to 120 min. Then, we placed the specimens labelled with both surgical and BM numbers into blue cassettes and washed them with running tap water for 2 h. Subsequently, they were placed in 10% buffered formalin until ready to load into processor. The primary antibody we used was mouse monoclonal anti-human Ang-1 (MAB923; Clone 171718; 5 mg ml^–1^; R&D Systems, Minneapolis, MN, USA). The working concentration was 25 *μ*g ml^–1^. IHC was executed as previously described (Seval *et al*, 2008). A score of 1–6 was calculated for each specimen according to the staining intensity (0=none, 1=weak, 2=strong) and the percentage of myeloid cells positively stained (1=0–25%, 2=26–50%, 3=51–75%, 4=76–100%) by the pathologists who were blind to the results of *Ang-1* mRNA expression. Erythroid cells were excluded from calculation.

### Cytogenetic study

Chromosomal analyses were performed on BM cells after 1−3 days of unstimulated culture. The metaphase cells were banded by trypsin-Giemsa technique and karyotyped in accordance with the International System for Human Cytogenetic Nomenclature as previously described (Tien *et al*, 1995).

### Statistical analysis

We used Mann–Whitney *U*-test to analyse the difference in the expression of *Ang-1*, *Ang-2*, *Tie2*, *VEGF-A* and *VEGF-C* between the MDS and control groups and between lower-risk and higher-risk MDS. Correlation between the *Ang-1* mRNA expression and protein expression by IHC was accessed by the Spearman's rank correlation. Overall survival was measured from the date of first diagnosis to death from any cause or the last follow-up. We adopted Kaplan–Meier estimation to plot survival curves, and used log-rank tests to examine the difference between groups. Hazard ratio (HR) and 95% confidence interval (CI) was estimated by Cox proportional hazards regression models to determine independent risk factors associated with overall survival in multivariate analyses. Two-sided *P*-values <0.05 were considered statistically significant. All statistical analyses were accomplished with the SPSS 17 (SPSS Inc., Chicago, IL, USA).

## Results

### Characteristics of the patients

Among the 208 MDS patients recruited (Table 1), 143 were males and 65 were females with a median age of 65 years (range, 14–88). According to the FAB classification (*n*=208), 77 patients had refractory anaemia (RA), 13 RA with ring sideroblasts (RARS), 81 RA with excess blasts (RAEB), 19 RAEB in transformation (RAEB-t) and 18 chronic myelomonocytic leukaemia. Compared with MDS patients in the United States (Rollison *et al*, 2008), our cohort had higher incidence of RAEB/RAEB-t (48.1% *vs* 15.5%, *P*<0.0001) but lower incidence of RARS (6.25% *vs* 11.6%, *P*=0.0147), similar to those in Korea, an Asian country (Lee *et al*, 2003). By the World Health Organization (WHO)-2008 classification (*n*=171), 36 patients had refractory cytopenia with unilineage dysplasia; 10 RARS; 41 refractory cytopenia with multilineage dysplasia; 81 RAEB-1 or RAEB-2; and three, MDS-unclassified.

One hundred and ninety-six patients had cytogenetic data, thus could be stratified by international prognosis scoring system (IPSS); 27 patients were in low, 93 in intermediate-1 (INT-1), 54 in INT-2 and 22 in high-risk groups.

### Comparison of angiogenic factor expression between MDS patients and normal controls

The expression of *Ang-1*, *Ang-2*, *Tie2*, *VEGF-A* and *VEGF-C* was quantified as a ratio with the expression of the housekeeping gene *RPLP0*. We found that the median levels of *Ang-1*, *Tie2*, *VEGF-A* and *VEGF-C* were significantly higher (*P*-value all <0.001; Supplementary Figure 1), but that of *Ang-2* was significantly lower (*P*=0.019) in MDS patients than in normal controls.

### Correlation between Ang-1 RNA expression and protein expression

**Figure 1:**
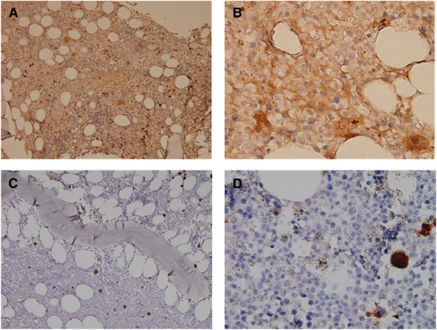
Representative IHC stainings of Ang-1 protein in BM biopsy specimens from two patients showing strong staining in one specimen from a patient with higher *Ang-1* mRNA expression (**A** and **B**), while weak staining in another specimen from a patient with lower mRNA expression (**C** and **D**). Megakaryocytes were served as positive internal control (magnification × 200 and × 1000, respectively).

Ang-1 protein expression measured by scoring of IHC correlated well with mRNA expression in the 40 patients studied (*P*=0.0057 by Spearman's rank correlation). Representative IHC of a sample with a higher score and another one with a lower score were demonstrated in Figure 1. Further, in order to directly demonstrate that BM blasts of MDS patients actually express Ang-1 protein, we prospectively sorted CD34^+^ cells from BM mononuclear cells by fluorescence activated cell sorter (FACSAria; BD Bioscience, San Jose, CA, USA) in two patients diagnosed as having RA with excess blasts and then performed immunocytochemical staining of these sorted CD34^+^ cells by immunoperoxidase technique. We showed that Ang-1 was strongly stained in these CD34^+^ cells (Supplementary Figure 2). It provided evidences that BM blasts might contribute to the Ang-1 protein expression in MDS patients.

### Association between BM angiogenic factor expressions and clinical features

Correlations between the expression of angiogenic factors and sex, age, hemogram, and FAB, WHO and IPSS subtypes were assessed. The *Ang-1* expression was much higher in FAB RAEB or RAEB-t subgroup than in RA or RARS subgroup; in WHO RAEB-1 or RAEB-2 subgroup than in other categories; and in IPSS INT-2/high-risk subgroup than in low/INT-1 subgroup (all *P*-value <0.001; Supplementary Figure 3). Furthermore, the *Ang-2* expression was also higher in INT-2/high than in low/INT-1 subgroup (*P*=0.03) but was not different among FAB or WHO subtypes (Supplementary Table 2). On the contrary, the *VEGF-A* expression was higher in FAB RA or RARS subgroup than in RAEB or RAEB-t subgroup (*P*=0.017) but was not different among WHO or IPSS subtypes. The expressions of *Tie2* and *VEGF-C* were similar between higher-risk and lower-risk subtypes by FAB, WHO or IPSS classification. There was no correlation between the expression of any one of the five angiogenic factors and sex and age. Regarding the hemogram, patients with normal platelet count (⩾10^5^ per *μ*l) had higher *VEGF-C* expression (*P*<0.001), and those with normal absolute neutrophil count (⩾1800 per *μ*l) had higher *VEGF-A* expression (*P*=0.029) (Supplementary Table 2).

### Association between BM angiogenic factor expressions and overall survival

**Figure 2:**
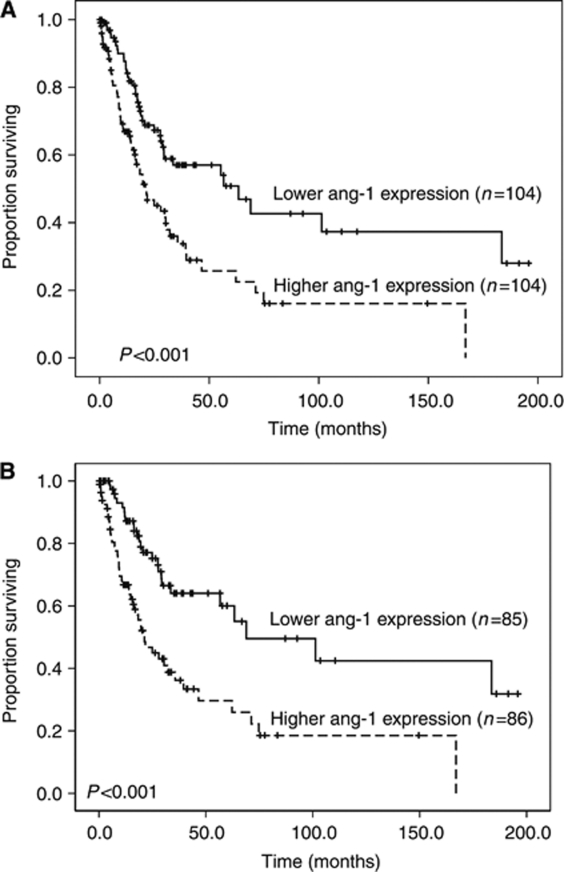
Kaplan–Meier curves of overall survival in all patients with newly diagnosed MDS according to (**A**) FAB and (**B**) WHO classifications stratified by the level of *Ang-1* expression (both log-rank test, *P*<0.001).

**Figure 3:**
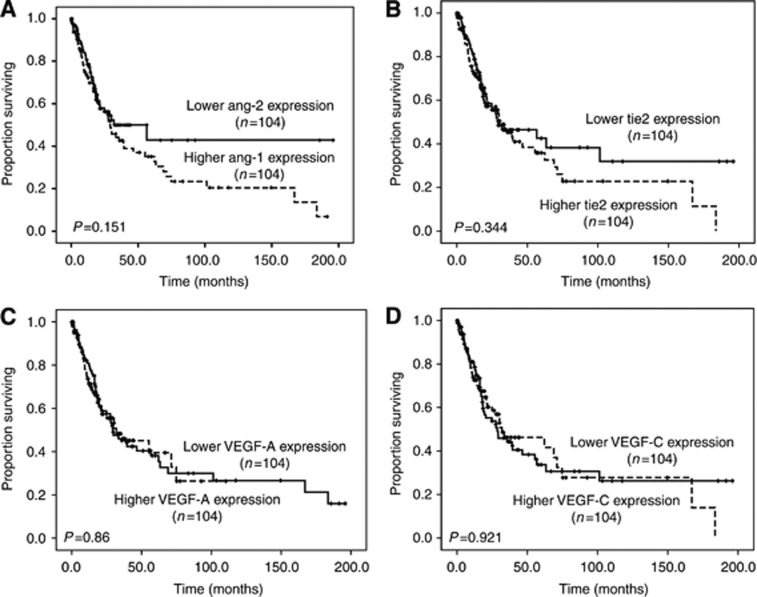
Kaplan–Meier curves of overall survival in all patients with newly diagnosed MDS stratified by the expression of (**A**) *Ang-2*, (**B**) *Tie2*, (**C**) *VEGF-A* and (**D**) *VEGF-C*, respectively. None had impact on prognosis.

The median value of each angiogenic factor expression was used as the cutoff level to divide patients into low- and high-expression groups. With a median follow-up time of 32.9 months (range, 0.03–196), the patients bearing higher *Ang-1* expression had higher frequency of disease transformation to acute leukaemia compared with those bearing lower *Ang-1* expression (31.5% *vs* 18.6%, *P*=0.023). Moreover, the patients with higher *Ang-1* expression had shorter overall survival than those with lower expression (median 20.8±4.5 months *vs* 63.3±17.8 months, *P*<0.001; Table 2; Figure 2). Similar result could be demonstrated in the 171 patients diagnosed according to WHO classification (median 21.3±3.8 months *vs* 68.9±27.3 months, *P*<0.001; Figure 2). The comparison of demographics between patients with lower and higher *Ang-1* expression was listed in Supplementary Table 3. Poor-risk cytogenetics occurred more frequently in the patients with higher *Ang-1* expression than in those with lower expression (24 out of 99 or 24.2% *vs* 11 out of 97 or 11.3%, *P*=0.025). There was no difference in other variables including sex, age, hemogram and treatment between the two groups. The expression of *Ang-2*, *Tie2*, *VEGF-A* and *VEGF-C* did not influence the clinical outcome (Table 2; Figure 3).

Variables including age, sex, karyotype, IPSS score and *Ang-1* expression were enrolled for the Cox proportional hazards multivariate analysis. The result distinctly identified higher *Ang-1* expression as an independent unfavourable prognostic factor for overall survival (HR=1.866; 95% CI, 1.204–2.892, *P*=0.005); along with other poor-risk factors, including higher IPSS score (INT-2 and high-risk subgroups) (HR=2.808, 95% CI, 1.796–4.389, *P*<0.001), poor-risk karyotype (HR=2.031, 95% CI, 1.221–3.377, *P*=0.006) and age ⩾60 years (HR=2.02, 95% CI, 1.266–3.222, *P*=0.003) (Table 3).

## Discussion

The present study showed that higher *Ang-1* expression in the BM is an independent poor prognostic factor for overall survival in MDS patients irrespective of age, karyotype and IPSS score. Additionally, patients having higher *Ang-1* expression would have higher chance of disease transformation to AML. To the best of our knowledge, this is the first report to demonstrate the prognostic implication of the Ang in MDS.

Relying on the work of the pioneer, Judah Folkman, and several successors, angiogenesis is proved to be fundamental to tumour growth and metastasis, including solid cancers and haematological malignancies (Folkman, 1971; Ellis and Fidler, 1996; Carmeliet and Jain, 2000; Hanahan and Weinberg, 2000; Hussong *et al*, 2000; Padro *et al*, 2000; Shih *et al*, 2009; Hou *et al*, 2010). MDS are considered to result from genetic and epigenetic aberrations, but the BM microenvironments including angiogenesis may also have a role (Aguayo *et al*, 2000; Tefferi and Vardiman, 2009). However, the clinical implication of angiogenesis in MDS remains unclear. Previous studies showed conflicting findings regarding the relationship of angiogenesis to clinical outcome in MDS patients. In one report, lower BM MVD was associated with significantly longer survival in univariate analysis, but it was not an independent prognostic factor by multivariate Cox regression analysis (Alexandrakis *et al*, 2005). In another study, decreased MVD was an independent predictor of longer progression-free survival, but not overall survival (Korkolopoulou *et al*, 2001), while in other studies, no prognostic role of MVD could be demonstrated (Lundberg *et al*, 2006; Madry *et al*, 2007).

Although the expression of VEGF family and the Ang/Tie2 receptor family correlates with clinical features and outcome in several solid cancers and haematological malignancies (Toi *et al*, 1994; O’Brien *et al*, 1995; Takahashi *et al*, 1995; Salven *et al*, 1998; Aguayo *et al*, 1999, 2002; Etoh *et al*, 2001; Tanaka *et al*, 2002; Sfiligoi *et al*, 2003; Chung *et al*, 2006), little is known about the prognostic implication of the expression of these angiogenic factors in MDS. It was reported that higher protein levels of VEGF in the BM were associated with shorter survival in MDS patients (Verstovsek *et al*, 2002). But only 41 MDS patients were recruited in that study. No prognostic significance of plasma VEGF levels in MDS patients was demonstrated in another report (Aguayo *et al*, 2002). Though Keith *et al* (2007) reported that the mRNA expression of *Ang-1*, *Ang-2* and the receptor *Tie2* in the BM was significantly higher in MDS patients, they did not show the data regarding prognosis. In this study, a large cohort of MDS patients was recruited to correlate the mRNA expression of five angiogenic factors including *VEGF-A*, *VEGF-C*, *Ang-1*, *Ang-2* and the receptor *Tie2* in the BM with the clinical outcome. The result exhibited that *Ang-1*, *Tie2*, *VEGF-A* and *VEGF-C* expression were higher, but *Ang-2* expression was lower in the BM of MDS patients compared with normal controls. The expression of *Ang-1* was consistently more elevated in higher-risk than in lower-risk subtypes of MDS by all three classification systems (Supplementary Table 2). In addition, higher expression of *Ang-1* was an independent unfavourable prognostic factor for overall survival by multivariate analysis, and was associated with a higher chance of transformation to AML.

**Figure 4:**
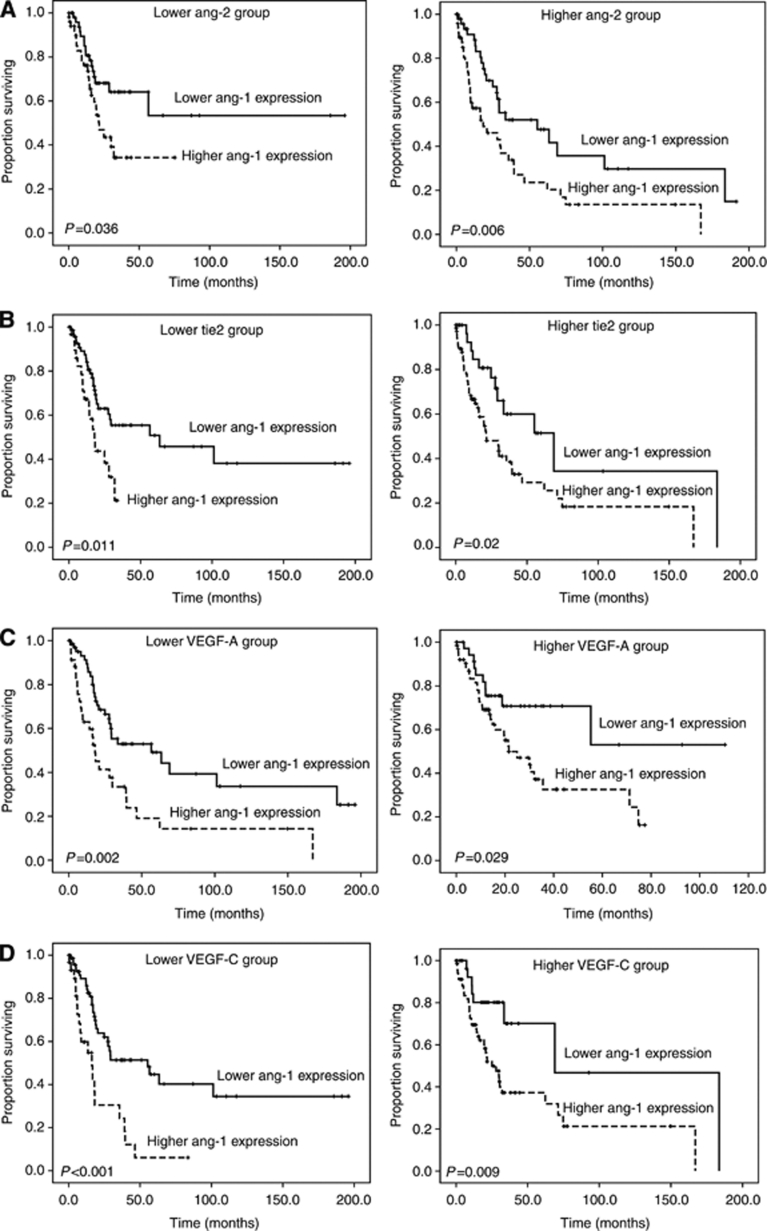
Subgroup analysis: Kaplan–Meier curves of overall survival stratified by *Ang-1* expression in the cohorts with low or high expression of (**A**) *Ang-2*, (**B**) *Tie2*, (**C**) *VEGF-A* and (**D**) *VEGF-C*. The significance of *Ang-1* expression for overall survival was not influenced by the levels of other angiogenic factors.

Like the VEGF family, the Ang/Tie2 signalling system is necessary for angiogenesis. The Ang family consists of Ang-1, 2, 3 and 4. They are extracellular ligands that specifically bind to a receptor tyrosine kinase, Tie2 on the surface of endothelial cells (Tait and Jones, 2004). Ang-1/Tie2 signalling cascade promotes the association of endothelium and pericytes (Armulik *et al*, 2005) and stimulates angiogenesis (Suri *et al*, 1998). Ang-1 regulates endothelial cell survival and maintains the integrity of vasculatures by autophosphorylation of Tie2 (Kim *et al*, 2000). Ang-2 does not result in activation of the receptor Tie2. It acts as a natural competitive inhibitor of Ang-1 (Maisonpierre *et al*, 1997). Without the presence of VEGF-A or other mitogenic factors, Ang-2 conveys a signal of vessel destabilisation, apoptosis and regression. In coordination with VEGF-A, however, it promotes proliferation and migration of endothelial cell, thereby inducing angiogenesis (Stratmann *et al*, 1998; Holash *et al*, 1999; Lobov *et al*, 2002). Thus, both Ang-1 and Ang-2 have pivotal, but different functions in the regulation of angiogenesis depending on the quantitative balance between these factors and other angiogenic factors. We previously demonstrated that higher expression of *Ang-2*, but not *Ang-1*, in the BM was a poor prognostic factor for overall survival in AML, particularly in the subgroup with high *VEGF-A* or *VEGF-C* levels (Hou *et al*, 2008). However, in this study, higher expression of *Ang-1*, but not *Ang-2*, predicted a poor outcome in MDS patients, no matter the levels of other angiogenic factors were high or low (Figure 4). The reason why the angiogenic factor that can predict the overall survival is different between MDS and AML is unclear. We and others showed *Ang-1*, *Ang-2* and *VEGF-A* expression levels were all increased in AML (de Bont *et al*, 2001; Watarai *et al*, 2002; Hou *et al*, 2008). However, in contrast to *Ang-1* and *VEGF-A*, which also showed higher expression in MDS patients than in normal controls, *Ang-2* expression was decreased in MDS patients (Supplementary Figure 1). Since coexpression of Ang-1 and VEGF-A enhances angiogenesis (Millauer *et al*, 1993; Suri *et al*, 1998; Ferrara *et al*, 2003), Ang-1 may have a more important role than Ang-2 in the pathogenesis of MDS.

There are some limitations in our study. First, BM biopsies were not done in all patients; hence, Ang-1 protein expression could not be measured by scoring of IHC in every case. However, we distinctly showed that there was a significant correlation between Ang-1 protein and mRNA expression in the 40 selected MDS patients. Second, the correlation between the expression of angiogenic factors and MVD were not done either. Recently, a dynamic contrast enhanced-magnetic resonance imaging (DCE-MRI) technique was developed to measure blood flow and perfusion in the BM of AML patients (Shih *et al*, 2009; Hou *et al*, 2010). Increased BM angiogenesis shown by this functional image foretold adverse outcome of AML patients. Further study to correlate the data of MVD, expression of angiogenic factors and DCE-MRI in the BM of MDS patients is warranted to have a better overview of the prognostic implication of angiogenesis in MDS.

To sum up, our study demonstrated that the expression of *Ang-1* was much more elevated in higher-risk than in lower-risk subtypes of MDS and was positively associated with disease transformation to acute leukaemia. The BM *Ang-1* expression was an independent prognostic factor for overall survival in MDS patients. It may serve as a new biomarker for foreseeing the clinical outcome and to stratify MDS patients for risk-adapted treatment. Inhibition of *Ang-1* expression may represent a therapeutic approach for patients with higher expression of this angiogenic factor.

## Figures and Tables

**Table 1:** The demographics of the 208 patients with myelodysplastic syndromes

**Variables**	**No. of patients (%)**
*Sex*	
Male	143 (69)
Female	65 (31)
Age (years)^a^	65 (14–88)
	
*FAB subtype*	208 (100)
RA	77 (37)
RARS	13 (6.3)
RAEB	81 (38.9)
RAEB-t	19 (9.1)
CMML	18 (8.7)
	
*WHO classification*	171 (100)
RCUD	36 (21)
RARS	10 (5.8)
RCMD	41 (24)
RAEB-1 or 2	81 (47.4)
MDS-U	3 (1.8)
	
*Karyotype* b	196 (100)
Good	125 (63.8)
Intermediate	36 (18.4)
Poor	35 (17.8)
	
*IPSS* c	196 (100)
Low	27 (13.8)
INT-1	93 (47.4)
INT-2	54 (27.6)
High	22 (11.2)

Abbreviations: FAB=French–American–British classification; RA=refractory anaemia; RARS=refractory anaemia with ring sideroblasts; RAEB=refractory anaemia with excess blasts; RAEB-t=refractory anaemia with excess blasts in transformation; CMML=chronic myelomonocytic leukaemia; RCUD=refractory cytopenia with unilineage dysplasia; RCMD=refractory cytopenia with multilineage dysplasia; MDS-U=myelodysplastic syndrome-unclassified; IPSS=international prognosis scoring system; INT=intermediate.

a Median (range).

b Good, normal karyotype, isolated -Y, del(5q) or del(20q); poor, complex (⩾3 abnormalities) or chromosome 7 anomalies; Intermediate, other abnormalities.

c International prognosis scoring system: low, 0; intermediate (INT)-1, 0.5–1; INT-2, 1.5–2; and high, ⩾2.5. The 196 patients with chromosome data could be stratified by this scoring system.

**Table 2:** Univariate analysis of the impact of angiogenic factor on overall survival in MDS patients

**Variable**	**No. of patients**	**Overall survival^a^**	***P*-value**
*Sex*			0.378
Male	143	28.7±5.5	
Female	65	62.2±20.2	
			
*Age (years)*			0.029
<60	83	68.9±23.4	
⩾60	125	27.9±4.1	
			
*Karyotype*			<0.001
Good/intermediate	161	39.2±11.1	
Poor	35	9.5±2.2	
			
*FAB classification*			<0.001
RA/RARS	90	68.9±10.3	
RAEB	81	18.3±2.4	
RAEB-t	19	14.8±3.2	
			
*IPSS*			<0.001
Low/INT-1	120	62.2±5.5	
INT-2/high	76	14.3±2.4	
			
*Ang-1*			<0.001
Low	104	63.3±17.8	
High	104	20.8±4.5	
			
*Ang-2*			0.151
Low	104	32±14.4	
High	104	29.3±4.1	
			
*Tie2*			0.344
Low	104	29.3±14.5	
High	104	30.4±5.4	
			
*VEGF-A*			0.860
Low	104	29.3±5.5	
High	104	32±12.1	
			
*VEGF-C*			0.921
Low	104	28.7±8	
High	104	32±13.8	

Abbreviations: MDS=myelodysplastic syndromes; FAB=French–American–British classification; RA=refractory anaemia; RARS=refractory anaemia with ring sideroblasts; RAEB=refractory anaemia with excess blasts; RAEB-t=refractory anaemia with excess blasts in transformation; IPSS=international prognosis scoring system; INT=intermediate; *Ang*=angiopoietin; *VEGF*=vascular endothelial growth factor.

a Median (months±s.d.).

Median value of each angiogenic factor was used as the cutoff level to define low- and high-expression groups.

**Table 3:** Multivariate analysis (Cox regression) of sex, age, karyotype, IPSS and *Ang-1* expression on the overall survival in MDS patients

**Variable**	**Hazard ratio**	**95% CI**	***P*-value**
Sex^a^	1.137	0.719–1.799	0.582
Age^b^	2.02	1.266–3.222	0.003
Karyotype^c^	2.031	1.221–3.377	0.006
IPSS^d^	2.808	1.796–4.389	<0.001
*Ang-1* e	1.866	1.204–2.892	0.005

Abbreviations: IPSS=international prognosis scoring system; *Ang-1*=angiopoietin-1; MDS=myelodysplastic syndromes; CI=confidence interval.

a Male *vs* female.

b Age ⩾60 years old *vs* age <60 years old.

c Poor karyotype *vs* good and intermediate karyotypes.

d Intermediate-2/high risk *vs* low risk/intermediate-1.

e Higher *Ang-1* expression *vs* lower *Ang-1* expression.
