# Supplementary material for: High bone marrow angiopoietin-1 expression is an independent poor prognostic factor for survival in patients with myelodysplastic syndromes
Source: Br J Cancer. 2011 Aug 30;105(7):975–82. doi: 10.1038/bjc.2011.340 (PMC3185953; doi:10.1038/bjc.2011.340)
Supplement: Supplementary Information [file bjc2011340x1.doc]

**Supplementary Table 1. Sequences of primer pairs and probes of angiogenic factors**

| Target gene | Forward (5’-3’) | Reverse (5’-3’) | Probe (5’-3’) |
| --- | --- | --- | --- |
| *Ang-2*  *Ang-1*  *Tie 2*  *VEGF-A*  *VEGF-C* | AAGAGATCAAGGCCTACTGTGACA  CAGACTGCAGAGCAGACCAGAA  CTGTGAAGGGCGAGTTCGA  CGAGGGCCTGGAGTGTGT  GAACACCAGCACGAGCTACCT | TCCTCACGTCGCTGAATAATTG  CTCTAGCTTGTAGGTGGATAATGAATTC  TGGTAGGAAGGAAGCTTGTTGAC  CCGCATAATCTGCATGGTGAT  CGGCAGGAAGTGTGATTGG | CCGCCTCCTCCAGCT  ACCCAGGTACTAAATCA  CAATCAGGATACGAACCATGA  CCCACTGAGGAGTCC  CAGCAAGACGTTATTTG |

**Supplementary Table 2. The correlation between five angiogenic factor expressions and clinical features**

| Variable | *Ang-1* | *P* value | *Ang-2* | *P* value | *Tie2* | *P* value | *VEGF-A* | *P* value | *VEGF-C* | *P* value |
| --- | --- | --- | --- | --- | --- | --- | --- | --- | --- | --- |
| Sex |  | 0.584 |  | 0.828 |  | 0.327 |  | 0.294 |  | 0.097 |
| Male | 0.024 (0-0.79) | | 0.032 (0-6.343) | | 0.0004 (0-0.039) | | 0.333 (0.008-29.304) | | 0.003 (0-0.449) | |
| Female | 0.027 (0.002-0.57) | | 0.028 (0.0002-164.289) | | 0.003 (0-0.05) | | 0.392 (0.023-64.276) | | 0.005 (0-0.204) | |
| Age, years |  | 0.459 |  | 0.962 |  | 0.381 |  | 0.165 |  | 0.351 |
| <60 | 0.023 (0-0.523) | | 0.031 (0-6.343) | | 0.0003 (0-0.018) | | 0.229 (0.017-29.304) | | 0.003 (0-0.449) | |
| ≥60 | 0.026 (0.0005-0.79) | | 0.032 (0.0005-164.289) | | 0.0004 (0-0.05) | | 0.392 (0.008-64.276) | | 0.003 (0-0.315) | |
| Hb (g/dL) |  | 0.066 |  | 0.771 |  | 0.168 |  | 0.909 |  | 0.666 |
| <10 | 0.027 (0-0.79) | | 0.032 (0-164.289) | | 0.0004 (0-0.05) | | 0.333 (0.008-64.276) | | 0.003 (0-0.449) | |
| ≥10 | 0.013 (0.001-0.295) | | 0.031 (0.0008-1.952) | | 0.0003 (1E-05-0.018) | | 0.353 (0.024-8.342) | | 0.002 (0.0001-0.081) | |
| PLT (/μL) |  | 0.102 |  | 0.923 |  | 0.358 |  | 0.271 |  | <0.001 |
| <10^5^ | 0.022 (0-0.523) | | 0.032 (0.0005-6.343) | | 0.0003 (0-0.015) | | 0.309 (0.0008-29.304) | | 0.002 (0-0.315) | |
| ≥10^5^ | 0.031 (0.001-0.79) | | 0.033 (0-164.289) | | 0.0004 (0-0.05) | | 0.351 (0.022-64.276) | | 0.006 (0-0.449) | |
| ANC (/μL) |  | 0.746 |  | 0.940 |  | 0.604 |  | 0.029 |  | 0.297 |
| <1800 | 0.022 (0-0.79) | | 0.033 (0-164.289) | | 0.0003 (0-0.05) | | 0.236 (0.012-29.304) | | 0.003 (0-0.142) | |
| ≥1800 | 0.027 (0.0005-0.4) | | 0.028 (0-6.343) | | 0.0004 (0-0.008) | | 0.49 (0.008-64.276) | | 0.003 (0-0.449) | |
| FAB |  | <0.001 |  | 0.221 |  | 0.551 |  | 0.017 |  | 0.051 |
| RA/RARS | 0.018 (0.0006-0.79) | | 0.023 (0.0002-6.343) | | 0.0004 (0-0.039) | | 0.6 (0.017-64.276) | | 0.004 (0-0.315) | |
| RAEB/RAEB-t | 0.045 (0-0.57) | | 0.039 (0-164.289) | | 0.0004 (0-0.05) | | 0.222 (0.008-29.304) | | 0.003 (0-0.449) | |
| WHO |  | <0.001 |  | 0.506 |  | 0.686 |  | 0.143 |  | 0.224 |
| Other categories | 0.018 (0.0006-0.79) | | 0.023 (0.0002-6.343) | | 0.0004 (0-0.039) | | 0.6 (0.017-64.276) | | 0.004 (0-0.315) | |
| RAEB-1/RAEB-2 | 0.047 (0-0.57) | | 0.032 (0-164.289) | | 0.0003 (0-0.05) | | 0.272 (0.008-29.304) | | 0.004 (0-0.449) | |
| IPSS |  | <0.001 |  | 0.03 |  | 0.199 |  | 0.174 |  | 0.052 |
| Low/INT-1 | 0.018 (0.0005-0.79) | | 0.018 (0-164.289) | | 0.0003 (0-0.05) | | 0.437 (0.012-64.276) | | 0.004 (0-0.449) | |
| INT-2/High | 0.041 (0-0.414) | | 0.043 (0-4.247) | | 0.0004 (0-0.015) | | 0.246 (0.008-29.304) | | 0.002 (0-0.315) | |
| The target gene expression was quantified as a ratio with expression of the housekeeping gene *RPLP0*.  Abbreviations: Hb, hemoglobin; PLT, platelet; ANC, absolute neutrophil count; FAB, French-American-British classification; RA, refractory anemia; RARS, refractory anemia with ring sideroblasts; RAEB, refractory anemia with excess blasts; RAEB-t, refractory anemia with excess blasts in transformation; WHO, World Health Organization-2008 classification, IPSS, international prognosis scoring system; INT, intermediate; *Ang*, angiopoietin; *VEGF,* vascular endothelial growth factor. | | | | | | | | | | |

**Supplementary Table 3. The comparison of demographics between MDS patients with lower and higher angiopoietin-1 expression**

| Lower expression (n=104) Higher expression (n=104) | | | |
| --- | --- | --- | --- |
| Variable | No. of patients | No. of patients | *P* value |
| Sex (M/F) | 74/30 | 69/35 | 0.55 |
| Age, years* | 63 (14-87) | 65 (16-88) | 0.565 |
| Laboratory tests* |  |  |  |
| Hemoglobin (g/dL) | 8.5 (4.4-14.4) | 8.1 (4.5-13.4) | 0.203 |
| Platelet count (K/μL) | 86.5 (3-471) | 92.5 (2-502) | 0.807 |
| Leukocyte count (K/μL) | 3.6 (0.94-79) | 3.99 (1.25-66.2) | 0.368 |
| Karyotype** |  |  | 0.025 |
| Good/Intermediate | 86 | 75 |  |
| Poor | 11 | 24 |  |
| Treatment |  |  | 0.697 |
| Intensive treatment*** | 14 | 17 |  |
| Supportive care  HSCT | 90  8 | 87  15 | 0.184 |
| * Median (range)  **A total of 196 patients, including 97 lower *Ang-1* expression and 99 higher *Ang-1* expression patients, had chromosome data at diagnosis.  *** Including the 8 patients with lower *Ang-1* expression and 15 with higher *Ang-1* expression who received allogeneic hematopoietic stem cell transplantation.  Abbreviations: MDS, myelodysplastic syndromes; HSCT, allogeneic hematopoietic stem cell transplantation. | | | |

Supplementary Figure 1

| A | B |
| --- | --- |
| C | D |
| E |  |

Supplementary Figure 2


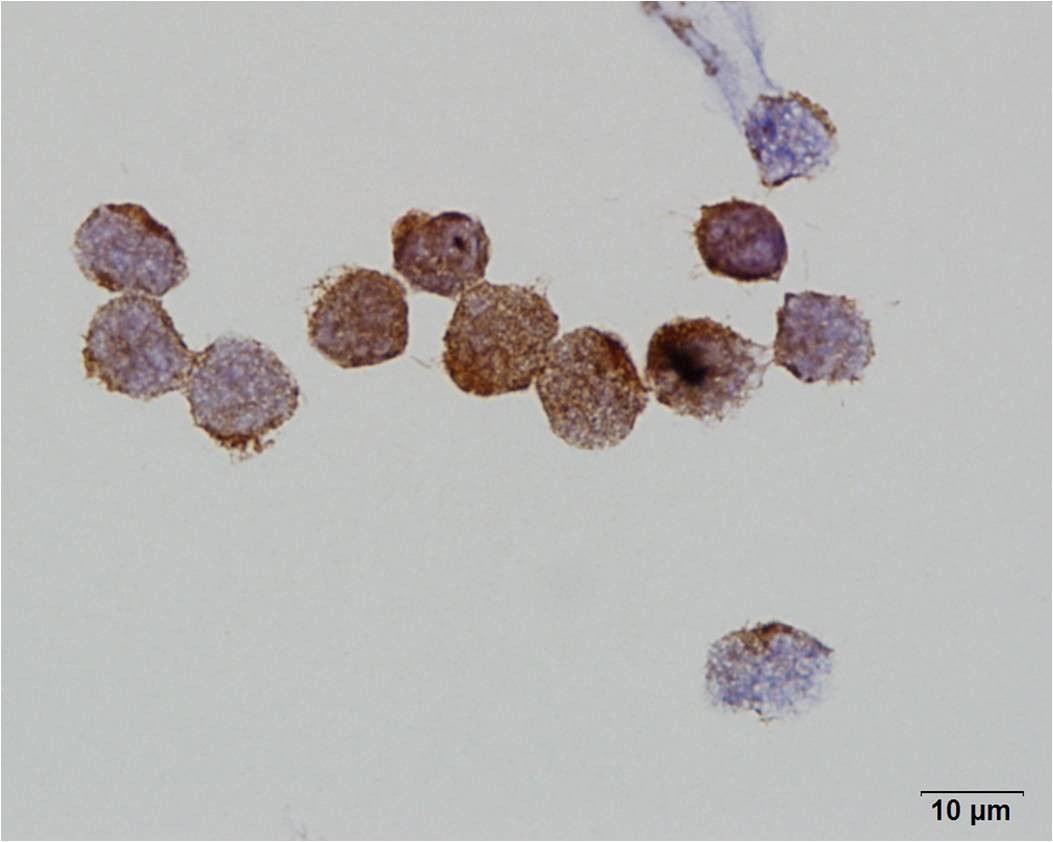


Supplementary Figure 3

| A |
| --- |
| B |
| C |

**Supplementary figure legend**

Supplementary Figure 1

Comparison of angiogenic factor expressions between MDS patients (P) and normal controls (N). *P* values were calculated using the Mann-Whitney *U* test. (A) angiopoietin-1 (*Ang-1*); (B) *Tie2*; (C) vascular endothelial growth factor A (*VEGF-A*); (D) *VEGF-C;* (E) angiopoietin-2 (*Ang-2*). The Y axis means target gene expression quantified as a ratio with expression of the housekeeping gene *RPLP0*.

Supplementary Figure 2

Representative immunocytochemical staining showing angiopoietin-1 protein expression in sorted CD34^+^ bone marrow cells from a patient with MDS. (Magnification 1000X)

Supplementary Figure 3

Comparison of angiopoietin-1 expression between patients with lower-risk and higher-risk MDS based on FAB, WHO and IPSS classifications. *P* values were calculated using the Mann-Whitney *U* test. (A) FAB RA/RARS *vs.* RAEB/RAEB-t (B) WHO Other categories *vs.* RAEB-1/RAEB-2 (C) IPSS Low/INT-1 *vs.* INT-2/High. The Y axis means target gene expression quantified as a ratio with expression of the housekeeping gene *RPLP0*.
